# New Alcohol Sensitivity in Patients With Post-acute Sequelae of SARS-CoV-2 (PASC): A Case Series

**DOI:** 10.7759/cureus.51286

**Published:** 2023-12-29

**Authors:** Ella F Eastin, Anushri Tiwari, Tom C Quach, Hector F Bonilla, Mitchell G Miglis, Phillip C Yang, Linda N Geng

**Affiliations:** 1 Medicine, Stanford University School of Medicine, Palo Alto, USA; 2 Medicine, Stanford University, Palo Alto, USA; 3 Neurology & Neurological Sciences, Stanford University School of Medicine, Palo Alto, USA

**Keywords:** alcohol sensitivity, alcohol reaction, alcohol intolerance, post-acute sequelae of sars cov-2 infection, long covid

## Abstract

Post-acute sequelae of SARS-CoV-2 (PASC), or long COVID, is characterized by persistent symptoms after acute SARS-CoV-2 infection that can vary from patient to patient. Here, we present a case series of four patients with a history of SARS-CoV-2 infection referred to the Post-Acute COVID-19 Syndrome (PACS) Clinic at Stanford University for evaluation of persistent symptoms, who also experienced new-onset alcohol sensitivity. Alcohol reactions and sensitivity are not well characterized in the literature as it relates to post-viral illness. While there have been some anecdotal reports of new alcohol sensitivity in PASC patients in the media, there is a paucity of published data in the medical literature about this topic. During their medical consultation, the patients self-reported new changes in their symptoms or behaviors following the use of alcohol. A new onset of alcohol sensitivities should be assessed along with other post-COVID-19 symptoms and may provide novel avenues to explore the pathobiology of illness and potential interventions.

## Introduction

Alcohol consumption can lead to different types of adverse reactions, ranging from mild to severe, from simple flushing to potentially life-threatening anaphylaxis. Alcohol intolerance typically describes an inherited genetic condition in which individuals - most common in East Asian populations - exhibit reduced capacities to metabolize ethanol due to alcohol dehydrogenase (ADH) and aldehyde dehydrogenase (ALDH) enzyme mutations [1-3]. Those affected may experience symptoms, including facial flushing, nausea, and low blood pressure, even at low levels of consumption. True alcohol allergy, a rare occurrence, is an immune response to alcohol compounds that leads to rashes, itchiness, swelling, severe stomach cramps, and potentially severe manifestations, such as anaphylaxis [3]. Toxic, irritant, or “hangover” effects of alcohol consumption can also occur. In this case series report, we use the non-specific term "alcohol sensitivity" to describe a range of adverse reactions to alcohol, including symptoms such as fatigue, brain fog, and headaches.

Symptoms following alcohol consumption have also been reported in those with Hodgkin's lymphoma [4], Epstein-Barr infection [5], and myalgic encephalomyelitis/chronic fatigue syndrome (ME/CFS) [6]. Patients with post-viral illnesses can experience a myriad of symptoms, such as persistent fatigue, headaches [7], tachycardia/palpitations, sleep disorders, and dyspnea. In a study on patients diagnosed with ME/CFS, a syndrome that can be triggered by viral infections, two-thirds of patients experience increased alcohol sensitivities leading to a reduction in alcohol intake [8]. Recent studies have also reported alcohol sensitivity after the onset of their illness in 65-80% of ME/CFS patients [9]. Individuals with a lowered tolerance, also known as acute sensitivity, may experience the effects of intoxication even after consuming relatively small amounts of alcohol.

Post-acute sequelae of SARS-CoV-2 (PASC), also known as long COVID or post-acute COVID-19 syndrome, describes new or worsening symptoms that can last for months and even years following acute COVID infection [10]. PASC has been described to encompass over a hundred other symptoms [10-13]. A subset of PASC has been compared to ME/CFS [12-15] due to the overlap in symptom presentation in patients with these syndromes. Mainstream media outlets have released articles [16-19] reporting on people developing a sensitivity to alcohol following a COVID infection, yet there is a paucity of medical literature to address this.

Alcohol sensitivity has been observed in a patient with PASC in a case report [20] but has not been widely studied. Here, we present a case series of patients reporting alcohol sensitivity following a SARS-CoV-2 infection that were evaluated at the Post-Acute COVID-19 Syndrome (PACS) Clinic at Stanford University. Patients had a history of confirmed COVID-19 infection and persistent post-COVID symptoms that lasted longer than four weeks. Patients 1, 2, and 4’s PASC symptoms persisted since their acute infection, while Patient 3’s PASC symptoms became noticeable two months after their initial COVID-19 infection. PASC symptoms and functional status were assessed during visits through clinical intake forms and a comprehensive medical evaluation during their consultation. Alcohol use habits were elicited, and any changes in drinking behaviors or symptoms following alcohol consumption were noted.

## Case presentation

Patient 1

A 60-year-old male with no prior medical history presented with five months of persistent symptoms following acute COVID infection, including headache, cognitive impairment, anxiety, and mood and sleep disturbances. These symptoms were disruptive to his regular activities including work and recreation. The patient reported a mild to moderate acute COVID infection that was managed outpatient with supportive care. Prior to his initial COVID infection, the patient reported consuming alcohol twice a month with no issue or reactions. The same amount of alcohol consumption post-COVID-19 now leads to headaches. The patient experienced chronic, daily headaches characterized by a squeezing sensation at the top and back of the head, typically worst at night. The patient had a normal head CT and brain MRI. He received four doses of COVID-19 vaccination, two before and two after infection.

Patient 2

A 40-year-old female, with a history of Ehlers-Danlos syndrome type III, asthma, anemia, hypotension, and migraines presented to the PACS clinic with over three months of persistent cognitive problems, dysautonomia symptoms, and shortness of breath suspected to be related to her initial acute COVID infection. Symptoms were also disruptive to regular daily activities. Prior to her initial COVID infection, she had no issues with alcohol tolerance and could easily tolerate about seven mixed drinks containing hard liquor in one night. A standard drink contains 14 grams of pure alcohol, with one standard drink equivalents including 5 ounces of wine or 12 ounces of beer [21]. After COVID infection, however, she reported feeling like she suffers from “alcohol poisoning” after drinking even small amounts of alcohol and feels “terrible” for several days after consumption. Her tolerance has decreased to the point where one beer would result in a severe “hangover,” along with exacerbation of PASC symptoms for three days thereafter. She received one dose of the vaccine before COVID infection.

Patient 3

A 49-year-old female, with a history of type 1 diabetes, celiac disease controlled by diet, and breast cancer presented to the PACS clinic 11 months after a COVID infection, reporting persistent symptoms of fatigue, paresthesias, shortness of breath, and decreased appetite. These symptoms were disruptive to the patient's regular activities. The patient used to consume several drinks per week and drink socially, but reported that she had not consumed alcohol for the last seven months due to decreased tolerance. The patient reported one instance, post-COVID infection, during which she had one glass of wine and had such a bad reaction that she felt she could not move. She described her symptoms as similar to a “bad hangover,” with a headache, grogginess, and “overwhelming” fatigue the next day. A week later, a single drink led to similar worsening of her symptoms. This patient received four doses of COVID-19 vaccination, three administered prior to her COVID infection and one post COVID infection. Notably, this patient also met the diagnostic criteria for ME/CFS.

Patient 4

A 36-year-old female, with a history of Raynaud’s syndrome, cryoglobulinemia, anxiety, Hashimoto’s thyroiditis, and sleep apnea, presented to the PACS clinic with symptoms of fatigue, unrefreshing sleep, brain fog, and hair loss approximately one year after her initial COVID infection, with significant functional debilitation and impact on basic activities of daily living. She also reported worsening anxiety and depression in addition to myalgias and arthralgias with numbness in her hands and feet. Prior to the patient's acute COVID infection, she drank socially without issue, but post-COVID infection, similar amounts of alcohol results in symptoms of flushing and headache. The patient had three doses of COVID-19 vaccine after her initial COVID infection.

## Discussion

This case series describes four PASC patients who developed new onset alcohol sensitivities after COVID-19 infection. The patients highlighted in this report, despite varying demographics and health backgrounds, share a new-onset sensitivity to alcohol post-COVID-19 infection, triggering unprecedented symptoms at similar or lower alcohol consumption levels. Responses to alcohol varied among patients. Some experienced individual symptoms like headaches or a delayed emergence of symptoms resembling a typical “hangover,” while others experienced a general worsening of their PASC symptoms. The alcohol sensitivity that is observed and reported from these patients generates interesting questions and hypotheses. It warrants further study as it may also reveal further insights into pathophysiology and provide guidance for lifestyle management in clinical care.

Alcohol sensitivity following viral infections in general have not been well characterized in the medical literature; however, it is a relatively common phenomenon observed in patients with ME/CFS, a related condition to PASC [11-13], and has been anecdotally reported on social media among PASC patients [15-18]. Observational data in a study of 114 diagnosed ME/CFS patients in the UK found that two thirds of the participants voluntarily decreased their alcohol consumption due to exacerbation of the following symptoms: fatigue, nausea, hangover, and sleep disturbances [8]. Studies have found that patients with ME/CFS - a post-viral condition - are more likely to report alcohol sensitivity and that patients with alcohol sensitivity experienced more overall different symptoms compared to those without reported sensitivity to alcohol [9]. It is not clear whether these alcohol reactions represent decreased tolerance threshold for alcohol, immune-mediated alcohol allergy to components in the alcohol drink, toxicity effects, or other sensitivity mechanisms. Considering the overlapping symptomatology between PASC and ME/CFS patients [14], it may be useful to consider mechanisms proposed in ME/CFS studies when studying alcohol intolerance patients with PASC. Orthostatic intolerance (OI) and autonomic dysfunction [13-15], neuroinflammation [22], gut microbiome changes [23-24], mitochondrial dysfunction (related to acetate metabolism) [25], and others have all been proposed and investigated as possible mechanisms for a number of ME/CFS symptoms, including alcohol intolerance.

OI is a condition characterized by an individual's inability to tolerate an upright posture because of an abnormal response of the body's autonomic nervous system to gravitational changes, resulting in inadequate blood flow to the heart and brain. This condition is notably common in patients with ME/CFS and is becoming increasingly recognized in patients with long COVID or PASC [13-15]. Our understanding of why individuals develop OI after viral illnesses is incomplete, but it is plausible that this could be a key mechanism by which alcohol consumption aggravates symptoms in those recovering from viral infections. Alcohol dilates blood vessels, potentially worsening the drop in blood pressure seen in those with OI. As a diuretic, alcohol may amplify dehydration in OI patients, further diminishing blood flow to the brain when upright and intensifying symptoms [26].

Recent observational studies have identified compositional changes within the gut microbiota to be associated with the presentation of in patients with PASC [23]. Although speculative, it is possible that alterations in the gut microbiome of PASC patients may alter the absorption of alcohol. Similarly, it has been reported that alcohol consumption can also alter the composition of the gut microbiome and increase permeability, allowing translocation of microbiota into circulation. This is proposed to cause inflammation at the liver and elsewhere in the body, including neuroinflammation [24]. Future investigations may explore if the altered microbiome in PASC patients, coupled with further alterations to the microbiome from alcohol, triggers exacerbation of immune-modulated responses through inflammatory markers, even at lower consumption levels. The presence of neuroinflammatory mediators in the pathogenesis of ME/CFS after viral infections [14] represents an additional mechanism that may contribute to increased sensitivity to alcohol in patients experiencing prolonged COVID symptoms. Neuroinflammation may also lead to reduced integrity of the blood-brain barrier [27], thus increasing sensitivities to not only alcohol but potentially other substances.

The mechanism behind alcohol sensitivity may be related to that seen in Hodgkin’s lymphoma or with medications, such as metronidazole [4,28]. Some suggest that prostaglandins (PGs) may have a role in acute or short-term alcohol sensitivity or adverse reaction based on the effectiveness of PG inhibitors on hangover and flushing reactions [29]. One case study [4] reporting on a patient with Hodgkin's lymphoma noted that the patient’s pain induced by alcohol consumption improved with ibuprofen. As ibuprofen inhibits the enzyme that produces prostaglandins, the authors suggest that lymph node vasodilation and prostaglandin production may lead to the observed, alcohol-related symptoms. It has been shown that SARS-CoV-2 infections are correlated with elevated prostaglandin E2 levels in the blood serum, with higher levels being associated with more severe infection [30]. If this prostaglandin elevation persists in some patients with PASC, then it may contribute to alcohol intolerance among these patients similar to pathways proposed in alcohol reactions seen in patients with Hodgkin’s lymphoma [4].

There are reports that suggest that alcohol tolerance and susceptibility to alcohol-related diseases may differ among racial and ethnic groups [1-3,31,32]. This case series is limited to four patients who self-identify as White or Hispanic, highlighting the need for further research investigating the potential influence of racial and ethnic background on alcohol intolerance in patients with PASC.

Current recommendations for the management of alcohol sensitivity include abstinence, avoidance, or the use of antihistamines to see if the severity of the reaction may be reduced [33]. Patients may be advised to avoid the type of drink or ingredient that may be triggering symptoms. The connection between differences in alcohol type and physiological effect is unclear, including whether it is the ethanol content itself or other compounds that are contained within various types of drinks, such as histamines and sulfites. More investigation is needed to understand the differences in response depending on the type of alcoholic beverage consumed, such as beer, hard liquor, or wine [34]. 

A definitive causal link between PASC and alcohol sensitivity cannot be established based on a limited case series. However, these cases reported here may reflect a larger population of individuals with PASC who suffer from new-onset alcohol sensitivity following COVID-19. Larger studies are needed across the diverse PASC patient populations and those who did not develop PASC after initial infection to ascertain the prevalence, manifestations, and durability of new alcohol sensitivity and determine its relationship to PASC and its illness course. Objective measures, such as blood alcohol levels, in future research on larger cohorts would also provide additional quantitative insight into the degree of alcohol reaction relative to ingested amount.

## Conclusions

New-onset alcohol reactions and sensitivity can occur after COVID-19 infection in patients with PASC. Clinicians assessing PASC patients should inquire about alcohol consumption and tolerance in their social history, as this information can provide insights into potential triggers for worsening symptoms and help guide lifestyle management strategies. Further research in the form of larger cohort studies is warranted to better understand the prevalence of this association in PASC patients and the range of alcohol reactions and sensitivities and explore potential associations with specific post-COVID-19 clinical phenotypes and other factors with post-COVID-19 alcohol sensitivity. Furthermore, investigating the underlying biological mechanisms responsible for new-onset alcohol reactions and sensitivity may provide valuable insights into the underlying pathophysiology of post-viral conditions, such as PASC and ME/CFS.
